# Combined pretreatment of sugarcane bagasse using alkali and ionic liquid to increase hemicellulose content and xylanase production

**DOI:** 10.1186/s12896-020-00657-4

**Published:** 2020-12-09

**Authors:** Rozina Rashid, Uroosa Ejaz, Firdous Imran Ali, Imran Ali Hashmi, Ahmed Bari, Jing Liu, Li Wang, Pengcheng Fu, Muhammad Sohail

**Affiliations:** 1 Department of Microbiology, University of Karachi, 75270, Karachi, Pakistan; 2 Department of Microbiology, University of Balochistan, Quetta, Pakistan; 3 Department of Chemistry, University of Karachi, 75270, Karachi, Pakistan; 4 Department of Pharmaceutical Chemistry, College of Pharmacy, King Saud University, Riyadh, Saudi Arabia; 5 State Key Laboratory of Marine Resource Utilization in South China Sea, Hainan University, Haikou, 570228 China

**Keywords:** *Bacillus aestuarii*, Ionic liquid, FTIR, NMR, Pretreatment,Xylan

## Abstract

**Background:**

Lignin in sugarcane bagasse (SB) hinders its utilization by microorganism, therefore, pretreatment methods are employed to make fermentable components accessible to the microbes. Multivariate analysis of different chemical pretreatment methods can aid to select the most appropriate strategy to valorize a particular biomass.

**Results:**

Amongst methods tested, the pretreatment by using sodium hydroxide in combination with methyltrioctylammonium chloride, an ionic liquid, (NaOH+IL) was the most significant for xylanase production by *Bacillus aestuarii *UE25. Investigation of optimal levels of five significant variables by adopting Box-Behnken design (BBD) predicted 20 IU mL^− 1^ of xylanase and experimentally, a titer of 17.77 IU mL^− 1^ was obtained which indicated the validity of the model. The production kinetics showed that volumetric productivity of xylanase was much higher after 24 h (833.33 IU L^− 1^ h^− 1^) than after 48 h (567.08 IU L^− 1^ h^− 1^). The extracted xylan from SB induced more xylanase in the fermentation medium than pretreated SB or commercially purified xylan. Nuclear Magnetic Resonance, Fourier transform infrared spectroscopy and scanning electron microscopy of SB indicated removal of lignin and changes in the structure of SB after NaOH+IL pretreatment and fermentation.

**Conclusion:**

Combined pretreatment of SB with alkali and methyltrioctylammonium chloride appeared better than other chemical methods for bacterial xylanase production and for the extraction of xylan form SB.

**Supplementary Information:**

The online version contains supplementary material available at 10.1186/s12896-020-00657-4.

## Background

Sugarcane bagasse (SB) is one of the abundant, low-cost agricultural deposits in the world which is mainly composed of lignin, hemicellulose, cellulose, wax and ash [1]. Owing to its site availability at sugar industries, SB can be used to generate additional revenues by utilizing it for the synthesis of chemicals, fuels and enzymes [2]. Pakistan, China, Mexico, India, Philippines, Thailand and Brazil are the major sugarcane producing countries [3] and therefore, can be befitted by adopting SB-based technologies. SB is a polysaccharides rich waste and hence a promising raw material in context of biorefineries [4] which can be utilized in various transformation processes. Therefore, the advances in biorefineries from SB have been investigated in numerous studies, with an immense range of configurations [5]. Sugar monomers like xylose and glucose can be obtained from SB which can be utilized in fermentation process to produce xylitol, lactic acid, ethanol, succinic acid, biopolymers, arabitol, electricity and antioxidants [3, 6].

Since presence of lignin and crystalline cellulose impedes microbial degradation of SB, therefore, pretreatment processes are universally adopted prior to utilization of the substrate in fermentation. A successful pretreatment or delignification enhances porosity, decreases polymerization and crystallinity of biomass, which elevates the accessibility of xylanases towards xylan [7]. Reports suggest that alkaline pretreatment of SB is better than acid (H_2_SO_4_) pretreatment as NaOH can dissolve more lignin in SB than H_2_SO_4_ [8]. Hydrogen peroxide [9] and ionic liquids (ILs) are other solvents that have also been employed for pretreatment of biomass. In comparison to other solvents, ILs show valuable properties including non-combustibility, lower toxicity and low vapor pressure [10, 11]. ILs convert crystalline components into amorphous forms that can easily be degraded because of the presence of more active sites. However, the selection of pretreatment methods depends on a number of factors including economic and environmental aspects. Ávila et al. [12] statistically compared the effectiveness of two pretreatment methods of SB namely, dilute acid and 1-ethyl-3-methylimidazolium acetate and utilized the pretreated substrate for the enzymatic hydrolysis to produce xylooligosacchaarides. Imidazolium-based ILs have been applied frequently, however, these are reportedly less biodegradable and harmful to the environment as well as to the aquatic organisms due to their toxic effects similar to chemicals (e.g., ammonia and phenol) used in disinfection processes [13]. Studies suggest that using such ILs under basic conditions may cause unexpected side reactions [14]. Therefore, an ammonium-based IL, methyltrioctylammonium chloride, was used in the current study. It is a water insoluble salt with low melting point and less viscosity [15]. The pretreatment of SB with this IL has been reported recently for the production of cellulases from thermophilic bacteria [15], therefore, it was tested to investigate its effectiveness in subsequent xylan extraction from SB. Considering the important component of lignocellulose (LC), xylan recovery from SB is a strategy to use hemicellulose component with commercial applicability. Current examples of industrial uses of xylan include in paper making, biomedical products, food products coating and packaging films [16]. The major limitation to the large scale hydrolysis of xylan is the cost of xylanase production. Xylan present in SB induces xylanase production [17], therefore, SB can serves as a promising feedstock for this purpose. In the current years, xylanase has acquired great attention due to its wide-ranging biotechnological uses. This enzyme is potentially applied to industries for bioconversion of hemicelluloses to xylose, clarification of juices and wines, de-inking processes of waste paper, textile and leather, refining the nutritional value of silage and fodder, agricultural waste treatment, paper and pulp industries, pharmaceuticals, ethanol and other useful substances [17]. Many of these processes employ thermostable xylanases that can withstand extreme process conditions.

Thermophiles are considered as cell factories for industrially important thermostable enzymes. The reactions by these enzymes at high temperature permit better solubility of reactants prompting faster hydrolysis [18]. *B. aestuarii* has previously been reported for the xylanase production by utilizing commercially available substrates [19]. To the best of our knowledge this is the first report on xylanase production by *B. aestuarii* using IL pretreated SB with the aid of a sequential and statistical optimization method. The main objective of the present study was to chemically pretreat SB in cost effective way so that lignin is removed and hemicellulosic content becomes available for fermentation purpose. Xylan was also extracted from pretreated SB and compared with commercially available substrate. The structural changes in SB after pretreatment and fermentation were recorded by nuclear magnetic resonance spectroscopy, Fourier transform infrared spectroscopy and scanning electron microscopy.

## Results

### Screening of thermophilic strains for xylanase production

Four thermophilic bacterial strains including *Aneurinibacillus thermoaerophilus *UE1, *Brevibacillus borstelensis *UE10, *Brevibacillus borstelensis *UE27 and *B. aestuarii *UE25 were initially screened for xylanase production by utilizing untreated and pretreated SB at different temperatures. *A. thermoaerophilus *UE1 did not produce any enzyme on hydrogen peroxide pretreated SB, however, it yielded the titers as high as 4.55 IU mL^− 1^ at 60 °C cultivation on other chemically treated SB (Supplementary Table S2) reflecting thermophilic nature of the strain. Likewise, *B. borstelensis *UE27 produced higher titers of xylanase at 60 °C than at 55 °C. *B. borstelensis *UE10 did not exhibit any xylanolytic activity and hence was found incapable of utilizing SB at mentioned temperatures. Nonetheless, *B. aestuarii *UE25 appeared as the most promising strain as it produced 18.38 IU mL^− 1^ at 60 °C. The strain was also cultivated at 37 °C to observe any effect of temperature on the xylanase production, however, a lower productivity (14.92 IU mL^− 1^) was noted (Supplementary Table S3) and hence the strain was cultivated at higher temperature in subsequent experiments.

### Evaluation of alkali and IL pretreatment of SB for xylanase production

The factors affecting xylanase production by *B. aestuarii *UE25 on pretreated SB were evaluated by a statistical method, PBD (Table 1). Among the three separate experimental designs performed, the data of the design 3 (for “H_2_O_2_/Untreated” SB) revealed that xylanase titers of 10.2 IU mL^− 1^ can be obtained using H_2_O_2 _pretreated SB (Table 2). However, this productivity was much lower than observed in response to the experiments performed for the design 2 (NaOH/H_2_SO_4_) and the design 1(IL/NaOH+IL). This observation may be attributed to the disadvantage of using H_2_O_2_ which causes lesser degree of delignification. The data obtained for “NaOH/H_2_SO_4_” pretreated SB revealed that the response was higher (more xylanase produced) from the experiments where alkali pretreated SB was used. The design for NaOH+IL/IL (pretreatment of SB by NaOH followed by IL or pretreatment by IL alone) was found to be the most striking as the highest titers of 37.48 IU mL^− 1^ of xylanase was obtained in one of the experiment where 5% inoculum was transferred to the medium of pH 7, containing 1% pretreated SB and MSM supplemented with 0.5% glucose and peptone and incubated for 24 h without agitation which indicated the effectiveness of using NaOH and IL for the pretreatment of SB.

**Table 1:** Plackett Burman experimental design (PBD) for xylanase production by using different pretreated sugarcane bagasse

Run Order	Bagasse pretreatment			Medium*	Incubation temperature (°C)	pH	Substrate concentration(%)	Inoculumsize (%)	Incubation time (h)	Agitation (150 rpm)
	PBD 1	PBD 2	PBD 3							
1	NaOH+IL^a^	NaOH^b^	H_2_O_2_^c^	MSM + 0.5% G	60	5	1	5	48	without
2	NaOH+IL	NaOH	H_2_O_2_	MSM + 0.5 G & P	55	7	1	5	24	without
3	IL^d^	H_2_SO_4_^e^	UTB^f^	MSM + 0.5 G & P	60	5	2	5	24	with
4	NaOH+IL	NaOH	H_2_O_2_	MSM + 0.5% G	60	7	1	10	24	with
5	NaOH+IL	NaOH	H_2_O_2_	MSM + 0.5 G & P	55	7	2	5	48	with
6	NaOH+IL	NaOH	H_2_O_2_	MSM + 0.5 G & P	60	5	2	10	24	without
7	IL	H_2_SO_4_	UTB	MSM + 0.5 G & P	60	7	1	10	48	with
8	IL	H_2_SO_4_	UTB	MSM + 0.5% G	60	7	2	5	48	without
9	IL	H_2_SO_4_	UTB	MSM + 0.5% G	55	7	2	10	24	without
10	NaOH+IL	NaOH	H_2_O_2_	MSM + 0.5% G	55	5	2	10	48	with
11	IL	H_2_SO_4_	UTB	MSM + 0.5 G & P	55	5	1	10	48	without
12	IL	H_2_SO_4_	UTB	MSM + 0.5% G	55	5	1	5	24	with

* ***MSM*** **Mineral salt medium,** ***G*** **Glucose,** ***P*** **Peptone**

**a = alkali + ionic liquid, b = alkali, c = hydrogen peroxide, d = ionic liquid, e = acid, f = untreated bagasse.**

**Table 2:** Xylanase response by Plackett Burman experimental design

Run order	Xylanase (IU mL^− 1^) obtained from different pretreated sugarcane bagasse		
	IL/NaOH+IL	NaOH/H_2_SO_4_	H_2_O_2_/UTB
1	10.72 ± 0.65	15.92 ± 1.95	10.2 ± 0.84
2	37.48 ± 2.7	4.96 ± 0.38	0
3	7.21 ± 0.07	11.4 ± 0.22	0
4	15.45 ± 0.56	0	0
5	19.26 ± 0.03	0	5.76 ± 0.58
6	9.32 ± 0.02	9.22 ± 0.09	0
7	4.92 ± 0.01	0	0
8	12.90 ± 0.04	6.64 ± 0.57	0
9	26.57 ± 0.09	6.29 ± 0.04	0
10	13.90 ± 0.04	5.94 ± 0.04	10.2 ± 0.35
11	14.87 ± 0.035	11.45 ± 0.04	7.71 ± 0.13
12	22.10 ± 0.12	15.92 ± 0.75	7.19 ± 0.04

***IL*** **Ionic liquid,** ***NaOH + IL*** **Alkali + ionic liquid,** ***NaOH*** **Alkali, H**_**2**_**SO**_**4=**_ **acid,** ***H***_***2***_***O***_***2***_ **Hydrogen peroxide,** ***UTB*** **Untreated bagasse.**

The regression analysis of the response (Supplementary Table S4), ANOVA (Supplementary Table S5) and Pareto chart (Supplementary Fig. S1) for the design 1 illustrated that five of the factors including inoculum size, incubation time, temperature, agitation and pH were significant for the xylanase production with *P*-value < 0.05. The analysis of the design 2 indicated four significant factors, including pretreatment, incubation period, pH and inoculum size (Supplementary Table S6, S7), while the design 3 revealed significance of three important factors namely pH, cultivation temperature and incubation period (Supplementary Table S8, S9). Nonetheless, one of these designs had to be selected for the optimization using response surface methodology. Since the xylanase titers obtained in the experiments suggested by the design 1 were apparently higher than the other designs therefore, it was hypothesized that the mean IU mL^− 1^ obtained for the design 1 was greater than the design 2 and the design 3. In order to statistically prove, the data from PBDs was compared by Two-way ANOVA. The model for NaOH+IL was found significant for pretreatment of SB and xylanase production with F-value >F-critical value and *P*-value < 0.05 for the columns (Supplementary Table S10).

### Optimization of xylanase production by fermention of NaOH+IL pretreated SB

The important factors influencing the xylanase production by *B. aestuarii* UE25 on NaOH+IL pretreated SB were further optimized by BBD through RSM approach. Five significant factors i.e. inoculum size, incubation time, temperature, agitation and pH (at three levels; − 1, 0, + 1) were studied in 46 experimental runs as proposed by BBD (Table 3). Xylanase titers acquired by BBD experiments were analyzed by ANOVA. The model presented an R^2^ value of 86.28% (Supplementary Table S11) indicating that the total variation of 86.28% was imputed to the factors in the model. Furthermore, the *p*-value (≤0.05) and F-value (17.29) indicated the significance of the model. A response optimization experiment was proposed by the software at optimum conditions of temperature, 59.8 °C; inoculum size, 7.03%; pH, 5; agitation, 141 rpm and incubation period, 24 h. Under these optimal conditions, 17.77 IU mL^− 1^ of xylanase titers were obtained, in comparison to the predicted value of 20 IU mL^− 1^ indicating the validity of the model. The results from the data showed that the *B. aestuarii *UE25 could produce optimal xylanase titers after 24 h into fermentation medium with pH 5 by inoculating 7.03% culture, incubating at 59.8 °C with agitation rate 141 rpm.

**Table 3:** Box-Behnken design for xylanase production by *Bacillus aestuarii *UE25

Run Order	Temperature (°C)	Incubation time (h)	pH	Agitation (rpm)	Inoculum size (%)	Xylanase production (IU mL^− 1^)^a^
1	55	24	6	75	7.5	0
2	60	24	6	75	7.5	14.20
3	55	72	6	75	7.5	7.13
4	60	72	6	75	7.5	9.15
5	57.5	48	5	0	7.5	6.69
6	57.5	48	7	0	7.5	17.95
7	57.5	48	5	150	7.5	13.32
8	57.5	48	7	150	7.5	11.79
9	57.5	24	6	75	5	11.70
10	57.5	72	6	75	5	9.59
11	57.5	24	6	75	10	12.41
12	57.5	72	6	75	10	12.76
13	55	48	5	75	7.5	0
14	60	48	5	75	7.5	6.74
15	55	48	7	75	7.5	6.86
16	60	48	7	75	7.5	9.33
17	57.5	48	6	0	5	10.12
18	57.5	48	6	150	5	20.17
19	57.5	48	6	0	10	30.10
20	57.5	48	6	150	10	12.76
21	57.5	24	5	75	7.5	7.74
22	57.5	72	5	75	7.5	8.09
23	57.5	24	7	75	7.5	8.36
24	57.5	72	7	75	7.5	7.39
25	55	48	6	0	7.5	11.03
26	60	48	6	0	7.5	14.96
27	55	48	6	150	7.5	11.53
28	60	48	6	150	7.5	22.26
29	57.5	48	5	75	5	8.85
30	57.5	48	7	75	5	8.80
31	57.5	48	5	75	10	9.76
32	57.5	48	7	75	10	6.92
33	55	48	6	75	5	0
34	60	48	6	75	5	16.54
35	55	48	6	75	10	15.84
36	60	48	6	75	10	12.76
37	57.5	24	6	0	7.5	11.35
38	57.5	72	6	0	7.5	16.90
39	57.5	24	6	150	7.5	8.69
40	57.5	72	6	150	7.5	16.66
41	57.5	48	6	75	7.5	6.87
42	57.5	48	6	75	7.5	8.09
43	57.5	48	6	75	7.5	5.87
44	57.5	48	6	75	7.5	7.92
45	57.5	48	6	75	7.5	8.62
46	57.5	48	6	75	7.5	7.50

^a^ With insignificant standard deviation

**Fig. 1:**
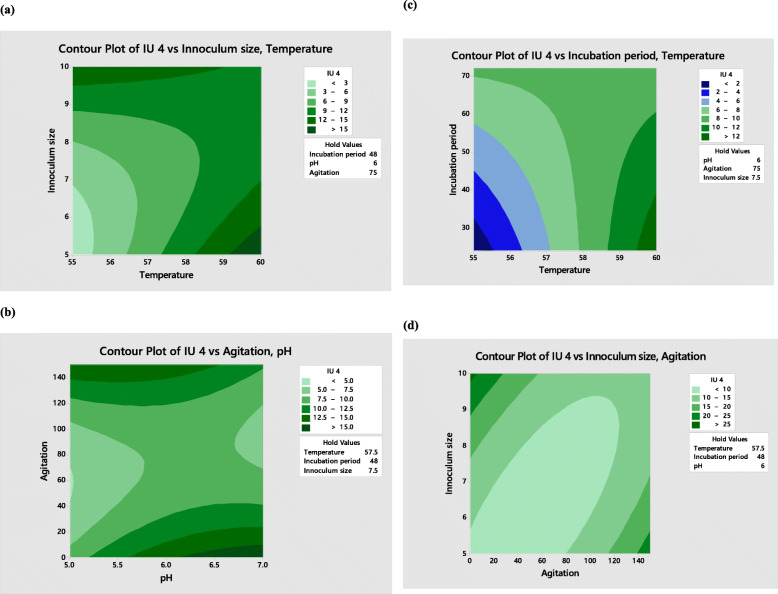
Contour Plot showing interaction of (**a**) Inoculum size and Temperature (**b**) Agitation and pH (**c**) Incubation period and Temperature (**d**) Inoculum size and Agitation on xylanase production by *Bacillus aestuarii* UE25

Interaction between the factors affecting xylanase production was studied against two independent variables and the optimal values were calculated by contour plots (Fig. 1). Interaction of temperature with pH was insignificant but the interaction of temperature and inoculum size was found to be statistically significant; when the inoculum size at 60 °C is kept 10%, xylanase production can be improved. The interactive effect of temperature and incubation time also appeared significant. Main effects indicate that increase in incubation time (up to 48 h) and temperature (60 °C) result in enhanced xylanase production. Xylanase titer increased with an increase in the inoculum size (up to 10%) when agitation rate was 140 rpm. A decrease in xylanase titer was observed when inoculum size was increased with pH, whereas agitation had a positive effect on xylanase production at pH 6.5–7.0.

### Kinetics of xylanase production

**Fig. 2:**
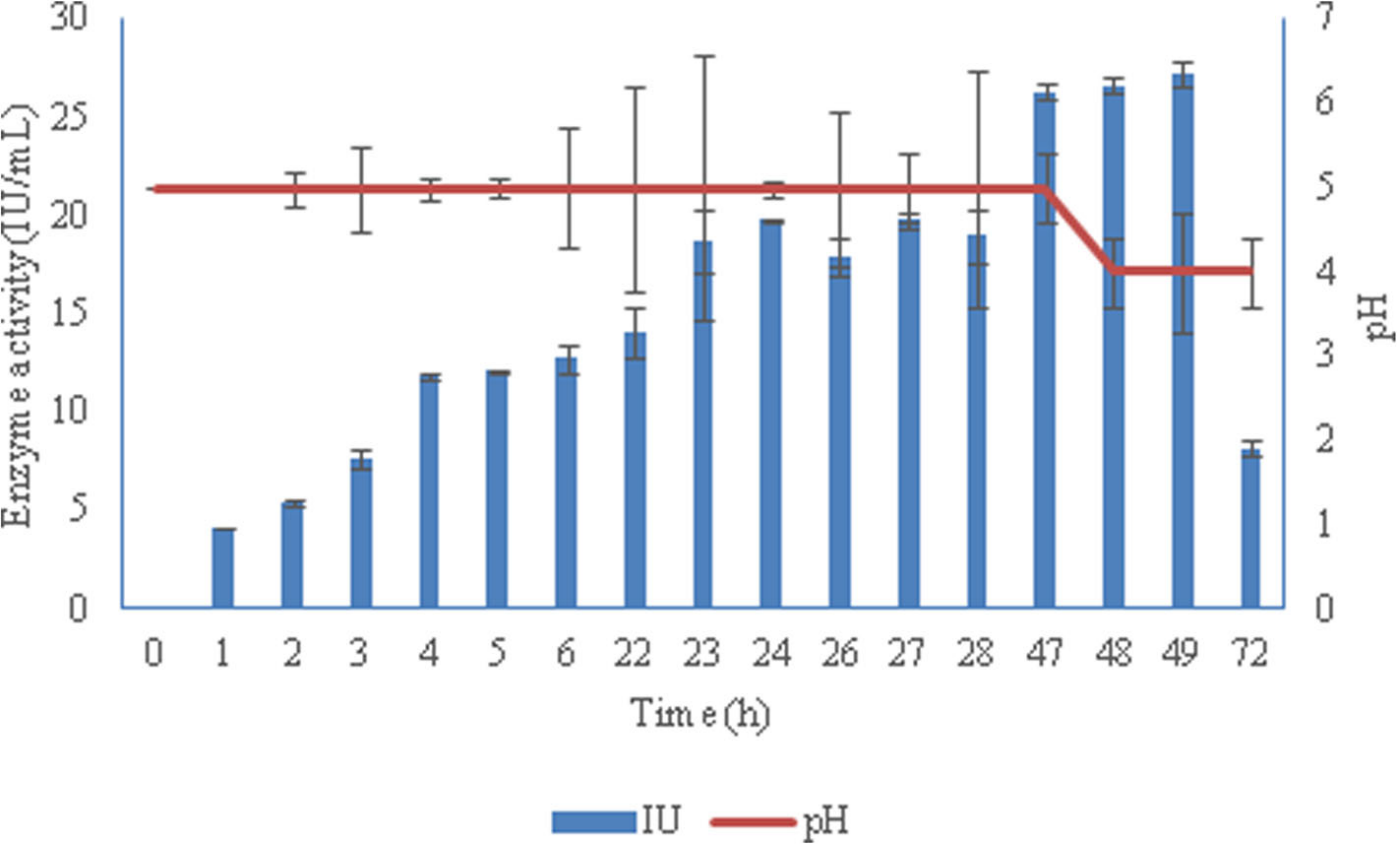
Time course study and effect of pH on production of xylanase by *Bacillus aestuarii *UE25

The results (Fig. 2) showed that *B. aestuarii *UE25 exhibited 20 IU mL^− 1^ after 24 h incubation and 27.22 IU mL^− 1^ after 49 h of incubation. To evaluate the titers obtained in 49 h in comparison to 24 h, productivity parameters were compared. The volumetric productivity (Q_p_) for xylanase was found to be 833.33 IU L^− 1^ h^− 1^ in 24 h and 567.08 IU L^− 1^ h^− 1^ in 48 h. Further increase in the fermentation time (72 h) resulted in decreased xylanase production. The drop in the yield of xylanase was probably due to the reduction of nutrients or proteolysis. Initially no change in pH was observed during cultivation, then after 48 h a drop in pH was noted that was coupled with a slight increase in the xylanase titers. Reduction in the medium pH was may be due to xylan hydrolysis (liberation of some monosaccharides causes the pH to reduce) [20] or acid produced during the fermentation process [21] and it indicated that the metabolism was not shifted towards proteolysis.

### Extraction of xylan from SB

Chemical composition of untreated sugarcane bagasse (used in this study) was investigated by Ejaz et al. [22] and presence of 30.8% xylan was reported in untreated sugarcane bagasse. In this study, xylan was extracted from NaOH+IL pretreated SB. Pretreated SB resulted in 45% yield of xylan whereas untreated SB resulted in 18.12% xylan. Higher xylanase production (1.25 fold) was noted after the fermentation of extracted xylan (from pretreated SB) than the commercially purified xylan (Table 4). Along with xylanases, EG, BGL and FPase assays were also performed. Increased IU mL^− 1^ for EG (from 21.95 to 36.06) and BGL (from 34.49 to 35.8) was obtained after 24 h of fermentation as compared to commercial xylan which indicated that cellulase and xylanase have the characteristics of co-expression.

**Table 4:** Xylanase and cellulase production by *Bacillus aestuarii* UE25 using extracted xylan

Substrate^a^	Enzyme productivity (IU mL^−1^)^a^							
	After 24 h				After 48 h			
	Xylanase	Endoglucanase	ß-glucosidase	Filterpaperase	Xylanase	Endoglucanase	ß-glucosidase	Filterpaperase
Extracted xylan from UTB	25.16 ± 0.02	36.06 ± 0.12	35.8 ± 0.04	5.47 ± 0.01	17.59 ± 0.05	18.71 ± 0.12	26.13 ± 0.04	9.86 ± 0.01
Extracted xylan from NaOH+IL TB	16.19 ± 0.04	22.67 ± 0.10	24.57 ± 0.06	6.52 ± 0.00	12.32 ± 0.04	23.12 ± 0.10	23.32 ± 0.02	7.00 ± 0.00
NaOH+IL TB	8.44 ± 0.16	32.92 ± 0.13	23.52 ± 0.02	7.92 ± 0.02	28.51 ± 0.06	67.42 ± 0.13	61.15 ± 0.06	6.04 ± 0.00
UTB	15.6 ± 0.49	12.34 ± 0.11	14.3 ± 0.01	7 ± 0.00	4.38 ± 0.00	22.14 ± 0.11	12.34 ± 0.01	2.23 ± .01
Xylan	20.24 ± 0.01	21.95 ± 0.31	34.49 ± 0.25	8.49 ± 0.01	13.02 ± 0.01	22.63 ± 0.31	18.91 ± 0.03	7.77 ± 0.03
CMC	14.78 ± 0.03	12.15 ± 0.05	30.57 ± 0.12	9.86 ± 0.03	15.66 ± 0.02	32.53 ± 0.03	35.67 ± 0.1	9.19 ± 0.02

^a^Xylan was extracted from untreated sugarcane bagasse (UTB) and alkali and ionic liquid pretreated (NaOH+IL TB)

### Scanning Electron microscopy

SEM images at 1000X magnification of the untreated SB samples (Supplementary Fig. S2a) showed highly ordered, compact and thick-walled fibers. Whereas, NaOH+ILpretreatment resulted in significant changes in structure of SB. SEM of pretreated SB sample (Supplementary Fig. S2b) shows that fibrils became more exposed by the removal of the superficial layer due to NaOH+IL pretreatment and the contact area was increased. It can be seen that the SmF of SB (Supplementary Fig. S2c) caused destruction and separation of fibers, which clearly showed the enzymatic degradation of SB.

### Fourier transform infrared (FTIR) spectroscopy analysis

**Fig. 3:**
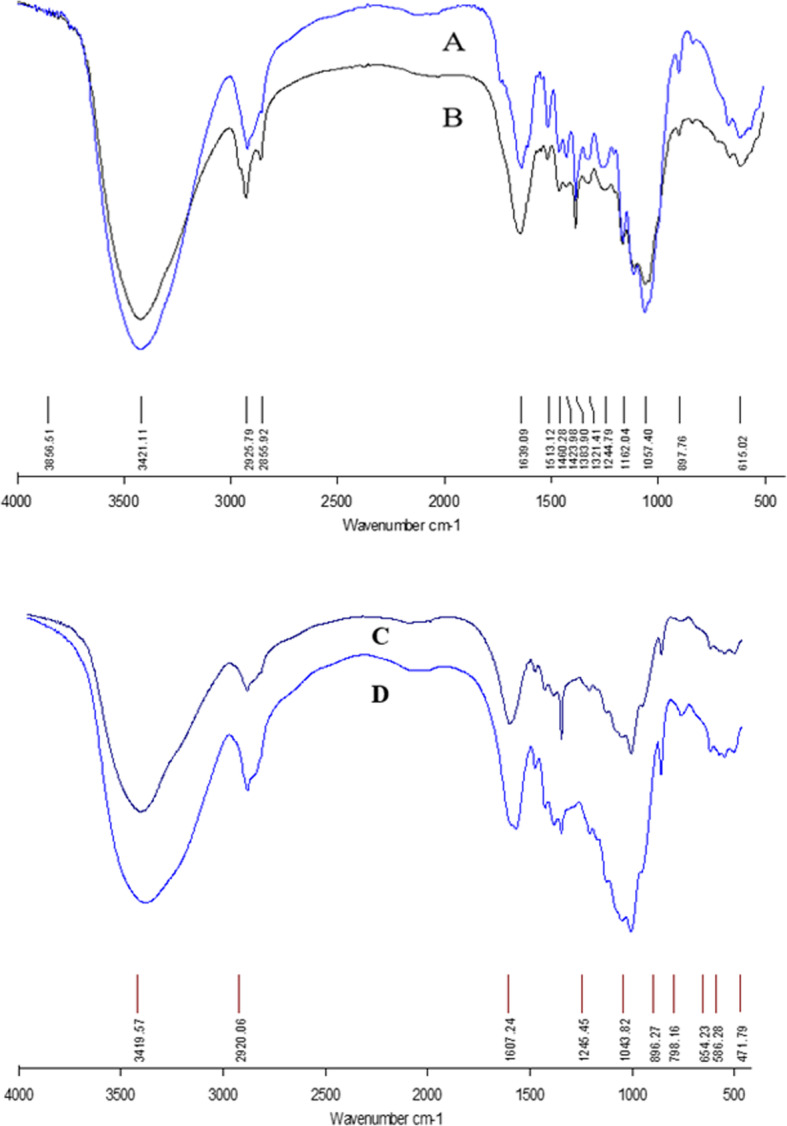
FTIR spectra of (**a**) Alkali and ionic liquid pretreated sugarcane bagasse (**b**) Fermented sugarcane bagasse (**c**) Extracted xylan from untreated SB (**d**) Extracted xylan from pretreated SB

FTIR spectra were studied for pretreated and fermented SB and the data was correlated with the untreated SB as given by Ejaz et al. [22]. In pretreated SB (Fig. 3a), the removal of lignin was evident by the changes in the region of 1260.93 cm^− 1^. The noticeable changes in the region associated with lignin from 1425.39 cm^− 1^ to 1511.93 cm^− 1^ [23] were also observed. The effect on lignin moiety was also visible by the change in the region of 3420.48 cm^− 1^. The FTIR spectra revealed presence of cellulose and hemicellulose components in pretreated SB by highlighting asymmetrical stretching of CH_2_ and CH of cellulose [24] at 2918.46 cm^− 1^ and the changes in the regions at 1109.77 cm^− 1^ and 1161.31 cm^− 1^ by hemicelluloses [25]. Moreover, β-glycosidic linkages between xylose units were observed at 897.90 cm^− 1^ [16].

The effects of fermentation on pretreated SB (Fig. 3b) were evident in the region between 1057 cm^− 1^ and 1162.04 cm^− 1^ that indicated the hydrolysis of hemicellulose and cellulose. A characteristic band at 897.76 cm^− 1^ for β-(1 → 4) glycosidic bond [26] was also present. The change in the stretching of C=O of hemicellulose and lignin was observed by the band at 1244.79 cm^− 1^ [27]. Presence of methoxy group due to acetyl portion of hemicellulose was visible by the band at 2855.92 cm^− 1^. The changes in the region of 2855.92 cm^− 1^ and 2925.79 cm^− 1^ revealed changes in cellulosic content of SB [24].

The structure of the extracted xylan from untreated (Fig. 3c) and pretreated (Fig. 3d) SB was also evaluated by FTIR analysis. The peaks at 3419, 2920, 1636, 1464, 1419, 1384, 1248, 1163, 1043 and 898 cm^− 1^ are assigned to hemicelluloses [27]. The C-O, C-C stretching or C-OH bending in the sugar units was observed as a sharp band at 1043 cm^− 1^, whereas, a sharp band at 896.27 cm^− 1^ presented the dominance of β-glycosidic linkages between the xylose units. A slight change was observed in the region of 894.84 cm^− 1^ for the untreated SB along with the absorption at 1636 cm^− 1^. The observation was further complemented by the spectra at 2920 cm^− 1^ and 2921 cm^− 1^ that were attributed to the C-H stretching vibrations caused by the water involved in the hydrogen bonding in xylan [22]. The quality of the extracted xylan was highlighted by the absence of absorbance at around 1720 cm^− 1^ that is otherwise observed if xylan is oxidized to ketone and carbonyls [28].

### Nuclear magnetic resonance (NMR) spectroscopy of SB

**Fig. 4:**
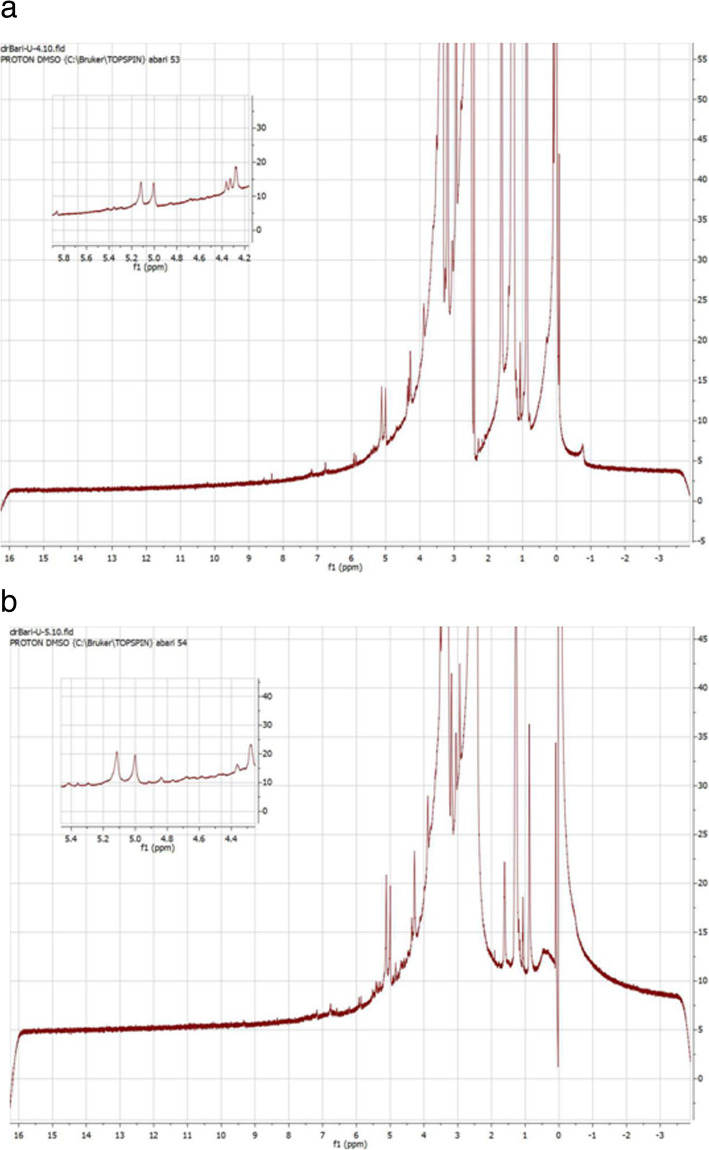
Nuclear Magnetic Resonance (NMR) spectra of (**a**) alkali and ionic liquid (NaOH+IL) pretreated SB (**b**) spectra of fermented SB

NMR spectroscopic technique is a rapid and sensitive technique to obtain information about small changes in certain chemical groups. ^1^H NMR spectroscopy technique was utilized to obtain information about the changes occurring in the SB samples before and after treatment with alkali and IL. The signals in the H NMR spectrum in the range of 6.0–6.5 are assigned to aromatic regions in lignin. The absence of aromatic proton peaks in NaOH+IL treated (Fig. 4a) and fermented (Fig. 4b) SB in the same range indicates the cleavage of β-O-4 linkage and decomposition or removal of the lignin part [29]. The signals in H NMR spectrum of untreated SB in the range 5.3 to 5.8 exhibit the Hβ in benzyl aryl ether which is a functional group present in lignin [30]. In contrast, spectra of pretreated SB do not show any signals in this region indicating degradation or removal of lignin in pretreated SB. The fewer peaks in the spectra of both the samples in the range 4.5–4.8 ppm show the absence of Hγ in β-O-4 aryl ether which is a characteristic of lignin [28]. The signals in the range of 3.0 to 4.5 ppm are assigned to the methoxyl protons. Moreover, the hydroxymethylene protons of sugars also display signals in the same range. The fermented and NaOH+IL pretreated bagasse did not show any peak in these regions. Similarly, the shifts at 4.1 and 4.4 ppm in both the samples represent the oxymethylene protons of xylose and arabinose residues.

## Discussion

Microbial xylanases have become one of the most explored enzymes due to their wide commercial applications, therefore, the search for new and novel strains and/or xylanase has always remained at the core of industrial biotechnology. Amongst the four strains investigated in this study, the xylanolytic potential of *B. borstelensis* [31] and *B. aestuarii* [19] have been reported previously, whereas, *A*. *thermoaerophilus* has not been described earlier as xylanase producer. Thermophilic nature of these strains was affirmed by their ability to produce more xylanase at higher temperatures that was in line with the findings by Chauhan et al. [19].

The aim of the study was to compare different pretreatment methods to SB and to adopt the most suitable method for xylanase production. The effectiveness of the pretreatment process can be assessed by enzyme titers [2]. Lignin is considered as a barrier that obstructs the access of enzymes thus, degradation of lignin may help cellulase and hemicellulases to act on their respective substrates [32]. Nerurker et al. [33] has previously studied the effect of different pretreatment methods for valorization of SB and the results proved that the chemical pretreatment valorized the SB and resulted in enhanced enzymatic saccharification.

In this study, the effectiveness of pretreatment methods was correlated with the titers of xylanase obtained from the fermentation of pretreated SB by *B. aestuarii *UE25. The factors affecting xylanase production on pretreated SB were evaluated by a statistical method, PBD, that was found to be an effective tool to improve the production of microbial enzymes [34–36].

De Guilherme et al. [9] discussed disadvantages of using H_2_O_2_ as it resulted in lesser degree of delignification and production of considerable foam during the reaction that caused the loss of solid matter. Similar observations were recorded in this study for H_2_O_2_ pretreatment.

Whereas, NaOH pretreatment was found better than H_2_SO_4_ pretreatment in terms of xylanase production. Acid pretreatment degrades the ultrastructure of LC waste by hydrolyzing xylan along with a portion of cellulose which leads to the loss of valuable sugars, while NaOH removes the lignin layer without removing the polysaccharides in SB and increases the accessibility of the enzymes to hemicelluloses. Qadir et al. [37] compared the effectiveness of acid and alkaline pretreatment of SB to utilize it for the simultaneous production of cellulase and xylanase by a yeast co-culture and statistically proved suitability of alkali pretreated SB as compare to acid pretreatment.

In this study, the data obtained from the design 1 of PBD revealed that pretreatment of SB by NaOH followed by IL (NaOH+IL) was found to be the most suitable pretreatment method for higher xylanase productivity. The NaOH+IL pretreatment of SB caused physical disruption of fibers and pith, the fibrils became more exposed by the removal of the superficial layer leading to structural alteration that interrupted the cross linking between cellulose, hemicellulose and lignin. Alkali primarily acts on lignin while IL modifies the crystalline shape of cellulose and hemicelluloses by breaking the hydrogen bonds in the structure of SB [38]. Methyltrioctylammonium consists of solvated chloride ions, therefore, has strong hydrogen-bonding basicity and is relatively more hydrophobic. In a previous report, quaternary ammonium IL was used for delignification of southern yellow pine wood at a high reaction temperature in dimethyl sulfoxide (DMSO) which is comparatively a toxic solvent [39]. In contrast, in this study the pretreatment was given at a lower temperature (70 °C); the use of ethanol instead of other more toxic solvent also makes this process greener. Moreover, the recyclability and reusability of IL is yet another advantage by which, the higher cost of IL can be compensated. Various techniques have been reported for recycling IL such as rotary evaporation [15], crystallization, liquid extraction, vacuum distillation, ion exchange nano-filtration, adsorption and salting out [40]. Ejaz et al. [41] recycled methyltrioctylammonium chloride for six times by evaporating ethanol from it retaining 70% xylanase productivity by recylcled IL pretreated SB. Hence, the effective pretreatment method for xylanase production was statistically confirmed by comparing the three designs by two-way ANOVA. It was observed that the model for design 1 was significant and ultimately was selected for further optimization through RSM.

RSM approaches furnish the actual value of every variable to be used optimally in a process [42]. Therefore, the significant factors (inoculum size, incubation time, temperature, agitation and pH) influencing xylanase production by utilizing NaOH+IL pretreated SB by*B. aestuarii* UE25 were investigated by BBD in RSM. Results showed that the best xylanase production was achieved when 7.03% inoculum size was used. As reported by Yoon et al. [43], an increase in inoculum size may initially enhance growth of the organism with subsequent decline in nutrients. Contrarily if lower inoculum size is transferred, organisms may put more efforts to colonize LC biomass resulting in a reduced enzyme production.

The effect of temperature on xylanase production by *B. aestuarii *UE25 was checked and optimal xylanase production was recorded at 59.8 °C. The thermophilic conditions significantly lessen the chance of media contamination by mesophilic microorganism and the storage time can be extended without losing enzyme activity [44]; therefore, studies on xylanase production by *B. aestuarii *UE25 warrant merit.

The change in pH causes alterations in ionic interaction among amino acids of an enzyme that results in loss of the conformation of the active site of the enzyme, therefore, an optimum pH is required to maintain the functional shape of the enzyme [45]. In our study, the results suggest that enzyme production was maximal at an acidic pH of 5. Researchers have found that breakdown of xylan in LC biomass is accelerated by acidic media which makes the substrate more sensitive and ultimately enzymes can work more efficiently [46]. Literature suggests that acidic xylanases have many potential industrial applications including in bio-bleaching, fruit juice clarification, or xylooligosachharides production [46].

The agitation of the fermentation medium was yet another significant factor. The amount of aeration and mixing of nutrients have been reported to affect the mass transfer and regulation of enzyme expression. In this study, maximum production of xylanase was recorded at an agitation of 141 rpm.

After analyzing the data and comparable response, an experimental design comprised of single run was proposed by the software. Experimental values (17.77 IU mL^− 1^) obtained under optimum conditions, in comparison to the predicted value (20 IU mL^− 1^) indicated the validity of the model. The enzyme production obtained under optimized conditions in BBD was lower than the enzyme production obtained in PBD. PBD is used to identify significant factors by evaluating linear effects and interactions of factors. While BBD allows to consider quadratic effects and provides an accurate value of each significant factor and, therefore, results obtained from BBD could be different from PBD [47].

Positive interaction was observed between the incubation time and temperature. The xylanase production by *B. aestuarii *UE25 was significantly higher with increase in incubation time and temperature adjusting the inoculum size up to 10%. However Yardimci and Cekmecelioglu [48] found a decrease in productivity with increase in inoculum size and reported 4% inoculum size for xylanase production.

Although the enzyme production kinetics showed an increasing pattern of xylanase production from 24 to 48 h, however the data for volumetric productivity (Q_p_) for xylanase illustrated that there was no significant increase in the xylanase titer in 48 h comparative to 24 h. Hence, it can be perceived that it is not cost effective to continue the cultivation until 48 h. Moreover, the design also presented 24 h as an appropriate duration for xylanase production. The duration of 24–48 h have previously been reported for xylanase production by Mrudula and Shyam [49] and Simphiwe et al. [50].

The SmF of xylan extracted from pretreated SB yielded 1.25 folds higher xylanase production than the commercially purified xylan after 24 h into fermentation. When the data for 48 h cultivation was compared, NaOH+IL pretreated SB was found to be the most appropriate substrate. In this study, 45% xylan was extracted from pretreated SB which is closely related to the results reported by Hauli et al. [51] who recovered 49% of xylan from SB by a similar method. It is also imperative to note that the extracted xylan has some cellulosic part and the co-expression of cellulase and xylanase has also been reported [52]. Therefore, production of EG, BGL and FPase was also detected. The data affirmed the cellulolytic nature of *B. aestuarii *UE25 [22] as much higher titers of cellulases were obtained. These findings assist the hypothesis stated by Han et al. [53] that a medium containing hemicelluloses gives rise to coordinated expression of cellulase and hemicellulase. The production of xylanase in cellulose containing medium indicates the presence of cellulose. It can also be attributed to the fact that ACEII, a cellulase regulator, also influences xylanase regulation [2]. Xylanase production reported in this study is significantly higher than the previous reports [37, 54]. Although, further improvement could be done by adopting other methods such as recombinant technology.

The SEM analysis of SB showed significant changes in the pretreated and fermented SB samples. Untreated samples showed rigid morphology of SB. Similar observation was reported by Chandel et al. [24]. Applying NaOH+IL pretreatment prior to enzymatic hydrolysis induced significant alteration in the structure of the SB, allowing an increased production of xylanase. Similar observations were reported by Ejaz et al. [55] where alkali and IL pretreatment resulted in destruction of SB structure. After SmF, the enzymatic degradation of SB was more prominent due to further destruction and separation of fibers making the structure more fragile [22].

The removal of lignin in pretreated SB was also confirmed by changes in the region 1260.93 cm^− 1^ and from 1425.39 cm^− 1^ to 1511.93 cm^− 1^ in FTIR spectra [23]. The enzymatic hydrolysis of cellulosic and hemicellulosic moieties was highlighted by the increased intensity at 1057 cm^− 1^ and 1162.04 cm^− 1^ [26]. Since SB is rich (20%) in arabinoxylan [56], therefore, a sharp band at 1043 cm^− 1^ [28] was visible in the spectra of extracted xylan from untreated SB. After xylan extraction from pretreated SB, high purity xylanfibers were obtained which demonstrate that the pretreatment and extraction protocol were suitable and efficient. Whereas, the band at 896.27 cm^− 1^ [23] was more intense in the pretreated SB which indicated that xylan yield was better from pretreated SB compared to untreated SB as only a slight change was observed in the region of 894.84 cm^− 1^ for the later substrate.

The NMR studies confirmed the lignin ratio in untreated bagasse in the range of 6.0–6.5 while the lower absorbance in these regions in NaOH+IL treated SB indicated the removal of the lignin part [29]. Similarly, the shifts at 4.1 and 4.4 ppm in pretreated samples are representing the oxymethylene protons of xylose and arabinose residues [28].

## Conclusions

From the findings of present study, it can be concluded that thermophilic bacterial strain (*Bacillus aestuarii* UE25) produced higher titers of xylanase by utilizing alkali and IL pretreated SB which is not reported earlier. Sequential pretreatment of SB with alkali followed by methyltrioctylammonium chloride was found statistically more significant than acid, alkaline or hydrogen peroxide pretreatment. The analyses (SEM, FTIR and NMR) also support the strategy to use IL for SB pretreatment as it is evident by structural changes in SB such as lignin removal and xylan accessibility. The presence of xylan extracted from SB in the production medium yielded higher xylanase productivity than that of commercially available xylan. It is expected that the present study will provide an opportunity for a sustainable sugarcane industry to use low-cost agro-waste (SB) for xylanase production by *B. aestuarii *UE25.

## Materials and methods

### Pretreatment of sugarcane bagasse

SB was provided by a local industry. Residual sugars were removed by excessive washing with tap water followed by drying at 60 °C for overnight up to 0% of moisture content. The dried substrate was ground to 300 μm mesh size using coffee bean grinder (Anex TS-630S). This untreated SB was used for different chemical pretreatments.

For alkaline and acid pretreatment, powdered SB was soaked in 1% (w/v) sodium hydroxide (NaOH) or sulfuric acid (H_2_SO_4_), respectively, at the rate of 50 ml g^− 1^ and left at room temperature for 24 h [37]. Any residual acid or alkali was neutralized by repeated washing with tap water. The pretreated SB was then oven dried at 60 °C for 24 h.

Hydrogen peroxide (H_2_O_2_) pretreatment of SB was performed by swamping 4% (w/v) aqueous suspension of SB in a 7.35% (v/v) hydrogen peroxide solution. The slurry was stirred using a magnetic stirrer at 100 rpm for 1 h at room temperature [9] and filtered with Whatman No. 1 filter paper. The remaining solid fraction was washed and stored at 4 °C.

Ionic Liquid (IL) pretreatment was carried out by loading SB in methytrioctylammonium chloride (Merck, Germany) at the ratio of 1:15 at 70 °C for 30 min. After filtration, washed thrice with ethanol and thrice with distilled water [22] and dried. For a combination of alkaline-ionic liquid (NaOH+IL) pretreatment, alkali pretreated SB was further pretreated with the IL as mentioned. Ethanol was evaporated from the reused IL and recycled IL was used without further purification [22].

### Bacterial strain and inoculum preparation

Initially four thermophilic strains, namely *Aneurinibacillus thermoaerophilus *UE1, *Brevibacillus borstelensis *UE10 and UE27 and *B. aestuarii *UE25 were screened for xylanase production. The strains were obtained from the culture collection of the department of Microbiology, University of Karachi, purified and maintained on Nutrient Agar (Oxoid, UK). The inoculum was prepared by transferring an isolated colony into Nutrient broth (Oxoid, UK) and incubated at 60 °C for 48 h at 150 rpm. It was transferred to the appropriate production medium by maintaining OD_600_ at 0.3.

### Screening of bacteria for xylanase production

Four thermophilic bacterial strains were screened for xylanase production as mentioned by Shariq and Sohail [54] with slight modification. The inoculum was transferred to mineral salt medium (MSM) [g L^− 1^ of K_2_HPO_4_ 1, (NH_4_)_2_SO_4_ 1, MgSO_4_.7H_2_O 0.02, CaCl_2_ 1, FeCl_3_ 0.02] which contained 1% untreated or pretreated SB and separately incubated at 50 °C, 55 °C or 60 °C for 48 h. After 48 h, the cells were harvested by centrifugation at 5000 x*g* for 10 min and supernatant was served as a source of crude xylanase preparation.

### Fermentation of alkali and IL pretreated SB by *B. aestuarii *UE25

Since *B. aestuarii *UE25 yielded higher titers of xylanase so it was selected to study the effect of various factors on its xylanase production under submerged fermentation (SmF) of SB. In the initial step of optimization, PBD was employed. Three separate PBDs (for NaOH/ H_2_SO_4_ pretreated SB, H_2_O_2_/untreated SB and IL/NaOH+IL pretreated SB), each consisting of 12 different experiments were generated using Minitab18 software. Eight factors (Supplementary Table S1, online material) were screened at two levels including temperature (55, 60 °C), pH (5, 7), medium (Mineral salt medium, MSM, with 0.5% glucose or MSM with 0.5% glucose and peptone), inoculum size (5, 10%), incubation time (24, 48 h), pretreatment (with chemical or untreated), substrate concentration (1% or 2%) and agitation (with or without shaking).

After completion of the experiments, cell-free culture suspensions (CFCS) were collected and assayed for xylanase activity and IU mL^− 1^ of the enzyme was taken as response. All the experimental runs of PBD were analyzed whereupon five factors showed up as significant variables (temperature, agitation, pH, incubation timeand inoculum size) affecting the production of xylanase under SmF of SB. Optimization of these significant factors at three levels (− 1, 0, + 1) was carried out by adopting Box-Behnken design (BBD) in Response surface method (RSM) approach. The experimental runs of 46 (as generated by Minitab 18 software) were carried out in triplicate and the mean values were used for analysis. To investigate kinetics of xylanase production, SmF of SB was carried out under optimized conditions and aliquots were withdrawn intermittently. After centrifugation, the CFCS was used to determine xylanase activity and pH. The data was used to determine volumetric productivity after 24 h and 48 h.

### Extraction of xylan

Xylan was extracted from untreated and NaOH+IL pretreated SB [51] and was used in the medium to produce xylanase by *B. aestuarii *UE25. Briefly, SB was immersed in 10% sodium hydroxide at the ratio of 1:10 at 60 °C for overnight with constant agitation and then steamed for 3 h at 100 °C. The suspension was centrifuged at 10,000 x*g* for 15 min and the pellet was acidified with12 N hydrochloric acid to adjust the pH as 5.0. Xylan was precipitated by adding 1.5 volume of 95% ethanol and separated by centrifugation at 6000 x*g* for 10 min. The extracted xylan was dried in a hot air oven at 55 °C for 4 h, weighed and stored at room temperature. The true recovery of xylan was calculated using the following formula as reported by Hauli et al. [51]:

True recovery (%) = dry weight of extracted xylan (g) / weight of the SB sample (g) × 100.

The extracted xylan was fermented by *B. aestuarii *UE25 and the results were compared with beechwood xylan (Sigma, Aldrich).

### Enzyme assays

The enzyme preparation was assayed for xylanase, endoglucanase, β-glucosidase and filter-paperase activities by adding 25 μL of crude enzyme to 25 μL (0.5%w/v) beechwood xylan, carboxymethyl cellulose (CMC), salicin (Sigma, Aldrich) or filter paper strip (of 1.5 × 3 mm), respectively. The reaction mixture was incubated in a water bath at 60 °C for 15 min. The reaction was stopped by adding 150 μL dinitrosalicylic acid (DNS) reagent and boiled for 5 min. After cooling on ice, 720 μL of distilled water was added. OD_540_ was measured against blank and compared with the calibration curve of glucose or xylose.

One International unit of the enzyme activity was defined as 1 μmol of glucose or xylose produced by 1 mL of the enzyme per min under the standard assay conditions.

### Analysis of structural changes in SB

Samples of untreated, NaOH+IL treated and fermented SB were analyzed at 10 kV using Analytical Scanning Electron Microscope JSM-6380 A, JEOL USA.

FTIR analysis of heat dried samples was carried out by taking spectra on JASCO FTIR-4200, whereas Proton Nuclear magnetic resonance (1H NMR) spectroscopy was performed by using Bruker AV-700 MHz spectrometer operating at 700 MHz in DMSO-d6 solutions. The ppm (δ) represents chemical shifts referenced to the signals relevant to DMSO d6.

## Supplementary Information

**Additional file 1: Table S1**. List of factors screened by Plackett-Burman design for xylanase production by *Bacillus aestuarii* UE25. **Table S2**. Screening of thermophilic bacteria for xylanase production by using sugarcane bagasse. **Table S3**. Screening of *Bacillus aestuarii* UE25 for xylanase production at 37 °C. **Table S4**. Regression coefficient and *P* values of xylanase by NaOH+IL Plackett-Burman Design. **Table S5**. Analysis of Variance for xylanase production by *Bacillus aestuarii* UE25 by NaOH+IL Plackett-Burman Design. **Table S6**. Regression coefficient and P values of xylanase by Akali/Acid Plackett-Burman Design. **Table S7**. Analysis of Variance for xylanase production by *Bacillus aestuarii* UE25 by Alkali/Acid Plackett-Burman Design. **Table S8**. Regression coefficient and P values of xylanase by H_2_O_2_/Untreated Plackett-Burman Design. **Table S9**. Analysis of Variance for xylanase production by *Bacillus aestuarii* UE25 by H_2_O_2_/Untreated Plackett-Burman Design. **Table S10**. Two-Way Analysis of Variance for comparison of different Plackett-Burman experimental designs for xylanase production. **Table S11**. Analysis of variance of Box-Behnken design for xylanase production by *Bacillus aestuarii* UE25. **Fig. S1**. Pareto Chart of the standardized effects showing the significant factors. **Fig**. **S2.** Scanning electron micrographs of various samples of SB (a) Untreated SB (b) NaOH+IL pretreated SB (c) Fermented SB with *Bacillus aestuarii* UE25.

## Data Availability

The data associated with this manuscript has been provided in a supplementary file available online. The raw data can be obtained from the corresponding author.

## Abbreviations

BBD: Box-behnken design
BGL: ß-Glucosidase
CFCS: Cell-free culture suspensions
CMC: Carboxymethyl cellulose
DMSO: Dimethyl sulfoxide
DNS: Dinitrosalicylic acid
EG: Endoglucanase
FPase: Filter-paperase
FTIR: Fourier transform infrared spectroscopy
H_2_O_2_: Hydrogen peroxide
H_2_SO_4_: Sulfuric acid
IL: Ionic liquid
LC: Lignocellulose
MSM: Mineral salt medium
NaOH: Sodium hydroxide
NaOH+IL: Alkaline-ionic liquid
NMR: Nuclear magnetic resonance
PBD: Plackett-burman design
RSM: Response surface method
SB: Sugarcane bagasse
SmF: Submerged fermentation
UTB: Untreated bagasse
