# Supplementary material for: Combined pretreatment of sugarcane bagasse using alkali and ionic liquid to increase hemicellulose content and xylanase production
Source: BMC Biotechnol. 2020 Dec 9;20:64. doi: 10.1186/s12896-020-00657-4 (PMC7724814; doi:10.1186/s12896-020-00657-4)
Supplement: Supplementary file 1 — Additional file 1: Table S1. List of factors screened by Plackett-Burman design for xylanase production by Bacillus aestuarii UE25. Table S2. Screening of thermophilic bacteria for xylanase production by using sugarcane bagasse. Table S3. Screening of Bacillus aestuarii UE25 for xylanase production at 37 °C. Table S4. Regression coefficient and P values of xylanase by NaOH+IL Plackett-Burman Design. Table S5. Analysis of Variance for xylanase production by Bacillus aestuarii UE25 by NaOH+IL Plackett-Burman Design. Table S6. Regression coefficient and P values of xylanase by Akali/Acid Plackett-Burman Design. Table S7. Analysis of Variance for xylanase production by Bacillus aestuarii UE25 by Alkali/Acid Plackett-Burman Design. Table S8. Regression coefficient and P values of xylanase by H2O2/Untreated Plackett-Burman Design. Table S9. Analysis of Variance for xylanase production by Bacillus aestuarii UE25 by H2O2/Untreated Plackett-Burman Design. Table S10. Two-Way Analysis of Variance for comparison of different Plackett-Burman experimental designs for xylanase production. Table S11. Analysis of variance of Box-Behnken design for xylanase production by Bacillus aestuarii UE25. Fig. S1. Pareto Chart of the standardized effects showing the significant factors. Fig. S2. Scanning electron micrographs of various samples of SB (a) Untreated SB (b) NaOH+IL pretreated SB (c) Fermented SB with Bacillus aestuarii UE25. [file 12896_2020_657_MOESM1_ESM.docx]

**Comparative analysis of chemical pretreatments of sugarcane bagasse for xylanase production by *Bacillus aestuarii* UE25**

Rozina Rashid^1^, Uroosa Ejaz^1^, Firdous Imran Ali^2^, Imran ali Hashmi^1^, Ahmed Bari^3^, Jing Liu^4^, Li Wang^4^, Pengcheng Fu^4^, Muhammad Sohail^1*^

^1^Department of Microbiology, University of Karachi, Karachi-75270, Pakistan

^2^Department of Chemistry, University of Karachi, Karachi-75270, Pakistan

^3^Department of Pharmaceutical Chemistry, College of Pharmacy, King Saud University, Saudia Arabia

^4^State Key Laboratory of Marine Resource Utilization in South China Sea, Hainan University, Haikou, 570228 China

^*^Author for all correspondence: [msohail@uok.edu.pk](mailto:msohail@uok.edu.pk) ORCID ID: 0000-0002-7208-9441

**Table S1. List of factors screened by Plackett-Burman design for xylanase production by *Bacillus aestuarii* UE25**

| Factors | Low (-) level | High (+) level |
| --- | --- | --- |
| Medium | MSM + 0.5% Glucose | MSM + 0.5% Glucose & peptone |
| Incubation temperature | 55°C | 60°C |
| pH of the medium | 5 | 7 |
| Amount of sugarcane bagasse | 1% | 2% |
| Inoculum size (%) | 5 | 10 |
| Incubation period (h) | 24 | 48 |
| Agitation | With | Without |

**With alkali-ionic liquid, acid, alkali and Hydrogen peroxide pretreated bagasse**

**Table S2. Screening of thermophilic bacteria for xylanase production by using sugarcane bagasse**

| Temperature  (⁰C) | Strain | Xylanase production (IU mL^-1^)* by using pretreated sugarcane bagasse | | | |
| --- | --- | --- | --- | --- | --- |
|  |  | NaOH+IL^a^ | H_2_SO_4_^b^ | NaOH^c^ | H_2_O_2_^d^ |
| 50 | *Aneurinibacillus thermoaerophilus* UE1 | 0 | 0 | 0 | 0 |
|  | *Brevibacillus borstelensis* UE10 | 0 | 0 | 0 | 0 |
|  | *Bacillus aestuarii* UE25 | 11.38 | 0 | 7.73 | 0 |
|  | *Brevibacillus borstelensis* UE27 | 0 | 0 | 0 | 0 |
| 55 | *Aneurinibacillus thermoaerophilus* UE1 | 0 | 0 | 0 | 0 |
|  | *Brevibacillus borstelensis* UE10 | 0 | 0 | 0 | 0 |
|  | *Bacillus aestuarii* UE25 | 14.18 | 10.12 | 11.62 | 8.43 |
|  | *Brevibacillus borstelensis* UE27 | 4.82 | 2.71 | 4.40 | 0 |
| 60 | *Aneurinibacillus thermoaerophilus* UE1 | 4.55 | 3.41 | 3.99 | 0 |
|  | *Brevibacillus borstelensis* UE10 | 0 | 0 | 0 | 0 |
|  | *Bacillus aestuarii* UE25 | 18.38 | 7.1 | 16.05 | 7.81 |
|  | *Brevibacillus borstelensis* UE27 | 7.28 | 3.14 | 4.81 | 3.02 |

*** The values represent the average of triplicate with insignificant standard deviation**

**a=alkali-ionic liquid pretreated bagasse, b=acid pretreated bagasse, c=alkali pretreated bagasse and d=Hydrogen peroxide pretreated bagasse**

**Table S3. Screening of *Bacillus aestuarii* UE25 for xylanase production at 37⁰C.**

| Sugarcane bagasse pretreated with | Xylanase production (IU mL^-1^) |
| --- | --- |
| NaOH+IL^a^ | 14.92±0.17 |
| H_2_SO_4_^b^ | 7.19±0.13 |
| NaOH^c^ | 12.04±1.1 |
| H_2_O_2_^d^ | 7.05±0.44 |

**(a=alkali-ionic liquid pretreated bagasse, b=acid pretreated bagasse, c=alkali pretreated bagasse and d=Hydrogen peroxide pretreated bagasse)**

**Table S4. Regression coefficient and P values of xylanase by NaOH+IL Plackett-Burman Design**

| Term | Effect | Coefficient Estimate | P-Value | Significance |
| --- | --- | --- | --- | --- |
| Bagasse pre-treatment | 2.927 | 0.627 | 0.102 | * |
| Medium | -1.430 | 0.627 | 0.337 | * |
| Temperature | -12.277 | 0.627 | 0.002 | significant |
| Ph | 6.410 | 0.627 | 0.015 | significant |
| Substrate concentration | -2.730 | 0.627 | 0.118 | * |
| Innoculum size | -4.107 | 0.627 | 0.047 | significant |
| Incubation | -6.927 | 0.627 | 0.012 | significant |
| Agitation | 4.837 | 0.627 | 0.031 | significant |

(NaOH+IL = alkali and ionic liquid pretreated bagasse)

**Table S5. Analysis of Variance for xylanase production by *Bacillus aestuarii* UE25 by NaOH+IL Plackett-Burman Design**

| Source | DF | Sum of square | Mean square | F-Value | P-Value |
| --- | --- | --- | --- | --- | --- |
| Model | 8 | 894.314 | 111.789 | 23.68 | 0.012 |
| Linear | 8 | 894.314 | 111.789 | 23.68 | 0.012 |
| Bagasse pretreatment | 1 | 25.696 | 25.696 | 5.44 | 0.102 |
| Medium | 1 | 6.135 | 6.135 | 1.30 | 0.337 |
| temperature | 1 | 452.150 | 452.150 | 95.78 | 0.002 |
| pH | 1 | 123.264 | 123.264 | 26.11 | 0.015 |
| %of bagasse | 1 | 22.359 | 22.359 | 4.74 | 0.118 |
| Innoculum size | 1 | 50.594 | 50.594 | 10.72 | 0.047 |
| Incubation | 1 | 143.936 | 143.936 | 30.49 | 0.012 |
| Agitation | 1 | 70.180 | 70.180 | 14.87 | 0.031 |
| Error | 3 | 14.162 | 4.721 |  |  |
| Total | 11 |  |  |  |  |

(NaOH+IL = alkali and ionic liquid pretreated bagasse)

**Table S6. Regression coefficient and P values of xylanase by Akali/Acid Plackett-Burman Design**

| Term | Effect | Coefficient Estimate | P-Value | Significance |
| --- | --- | --- | --- | --- |
| Bagasse pretreatment | -1.142 | 0.179 | 0.050 | significant |
| Medium | -0.979 | 0.179 | 0.072 | * |
| Incubation temperature | 0.055 | 0.179 | 0.887 | * |
| pH | -4.174 | 0.179 | 0.001 | significant |
| % of Bagasse | -0.568 | 0.179 | 0.211 | * |
| Inoculum size | -1.675 | 0.179 | 0.018 | significant |
| Incubation period | -0.490 | 0.179 | 0.265 | significant |
| Agitation | 1.933 | 0.179 | 0.012 | * |

**Table S7. Analysis of Variance for xylanase production by *Bacillus aestuarii* UE25 by Alkali/Acid Plackett-Burman Design**

| Source | DF | Sum of square | Mean square | F-Value | P-Value |
| --- | --- | --- | --- | --- | --- |
| Model | 8 | 80.3631 | 10.0454 | 26.10 | 0.011 |
| Linear | 8 | 80.3631 | 10.0454 | 26.10 | 0.011 |
| Bagasse preatratment | 1 | 3.9121 | 3.9121 | 10.16 | 0.050 |
| Medium | 1 | 2.8730 | 2.8730 | 7.46 | 0.072 |
| Incubation temperature | 1 | 0.0092 | 0.0092 | 0.02 | 0.887 |
| pH | 1 | 52.2571 | 52.2571 | 135.77 | 0.001 |
| % of Bagasse | 1 | 0.9681 | 0.9681 | 2.52 | 0.211 |
| Inoculum size | 1 | 8.4141 | 8.4141 | 21.86 | 0.018 |
| Incubation period | 1 | 0.7195 | 0.7195 | 1.87 | 0.265 |
| Agitation | 1 | 11.2101 | 11.2101 | 29.13 | 0.012 |
| Error | 3 | 1.1546 | 0.3849 |  |  |
| Total | 11 |  |  |  |  |

**Table S8. Regression coefficient and P values of xylanase by H_2_O_2_/Untreated Plackett-Burman Design**

| Term | Effect | Coefficient Estimate | P-Value | Significance |
| --- | --- | --- | --- | --- |
| Bagasse pretreatment | 0.939 | 0.197 | 0.097 | * |
| Medium | -1.177 | 0.197 | 0.058 | * |
| Incubation temperature | -1.721 | 0.197 | 0.022 | significant |
| pH | -2.463 | 0.197 | 0.008 | significant |
| % of Bagasse | -0.762 | 0.197 | 0.149 | * |
| Inoculum size | -0.438 | 0.197 | 0.348 | * |
| Incubation period | 2.224 | 0.197 | 0.011 | significant |
| Agitation | -0.436 | 0.197 | 0.350 | * |

**Table S9. Analysis of Variance for xylanase production by *Bacillus aestuarii* UE25 by H_2_O_2_/Untreated Plackett-Burman Design**

| Source | DF | Sum of square | Mean square | F-Value | P-Value |
| --- | --- | --- | --- | --- | --- |
| Model | 8 | 51.6102 | 6.4513 | 13.83 | 0.027 |
| Linear | 8 | 51.6102 | 6.4513 | 13.83 | 0.027 |
| Bagasse pretreatment | 1 | 2.6461 | 2.6461 | 5.67 | 0.097 |
| Medium | 1 | 4.1595 | 4.1595 | 8.91 | 0.058 |
| Incubation temperature | 1 | 8.8838 | 8.8838 | 19.04 | 0.022 |
| pH | 1 | 18.1917 | 18.1917 | 38.99 | 0.008 |
| % of Bagasse | 1 | 1.7442 | 1.7442 | 3.74 | 0.149 |
| Inoculum size | 1 | 0.5742 | 0.5742 | 1.23 | 0.348 |
| Incubation period | 1 | 14.8408 | 14.8408 | 31.81 | 0.011 |
| Agitation | 1 | 0.5699 | 0.5699 | 1.22 | 0.350 |
| Error | 3 | 1.3998 | 0.4666 |  |  |
| Total | 11 |  |  |  |  |

**Table S10. Two-Way Analysis of Variance for comparison of different Plackett-Burman experimental designs for xylanase production**

| *Source of Variation* | *SS* | *df* | *MS* | *F* | *P-value* | *F crit* |
| --- | --- | --- | --- | --- | --- | --- |
| Rows | 468.0564 | 10 | 46.80564 | 1.155755 | 0.3733 | 2.347878 |
| Columns | 1151.215 | 2 | 575.6077 | 14.21327 | 0.000144 | 3.492828 |
| Error | 809.958 | 20 | 40.4979 |  |  |  |
|  |  |  |  |  |  |  |
| Total | 2429.23 | 32 |  |  |  |  |

**Table S11. Analysis of variance of Box-Behnken design for xylanase production by *Bacillus aestuarii* UE25**

| Source | DF | Sum of square | Mean square | F-Value | P-Value |
| --- | --- | --- | --- | --- | --- |
| Model | 12 | 1187.96 | 98.997 | 17.29 | 0.000 |
| Linear | 5 | 254.22 | 50.844 | 8.88 | 0.000 |
| Temperature | 1 | 179.31 | 179.312 | 31.32 | 0.000 |
| Incubation period | 1 | 10.91 | 10.913 | 1.91 | 0.177 |
| pH | 1 | 16.42 | 16.423 | 2.87 | 0.100 |
| Agitation | 1 | 0.23 | 0.230 | 0.04 | 0.842 |
| Innoculum size | 1 | 47.34 | 47.341 | 8.27 | 0.007 |
| Square | 3 | 571.99 | 190.664 | 33.31 | 0.000 |
| pH*pH | 1 | 26.36 | 26.356 | 4.60 | 0.039 |
| Agitation*Agitation | 1 | 418.34 | 418.343 | 73.08 | 0.000 |
| Innoculum size*Innoculum size | 1 | 114.74 | 114.739 | 20.04 | 0.000 |
| 2-Way Interaction | 4 | 361.75 | 90.439 | 15.80 | 0.000 |
| Temperature*Incubation period | 1 | 37.06 | 37.064 | 6.47 | 0.016 |
| Temperature*Innoculum size | 1 | 96.28 | 96.275 | 16.82 | 0.000 |
| pH*Agitation | 1 | 40.89 | 40.890 | 7.14 | 0.012 |
| Agitation*Innoculum size | 1 | 187.53 | 187.526 | 32.76 | 0.000 |
| Error | 33 | 188.90 | 5.724 |  |  |
| Lack-of-Fit | 28 | 184.04 | 6.573 | 6.76 | 0.021 |
| Pure Error | 5 | 4.86 | 0.972 |  |  |
| Total | 45 |  |  |  |  |

Model Summary

| S | R-sq | R-sq(adj) | PRESS | R-sq(pred) |
| --- | --- | --- | --- | --- |
| 2.39255 | 86.28% | 81.29% | 385.505 | 72.00% |


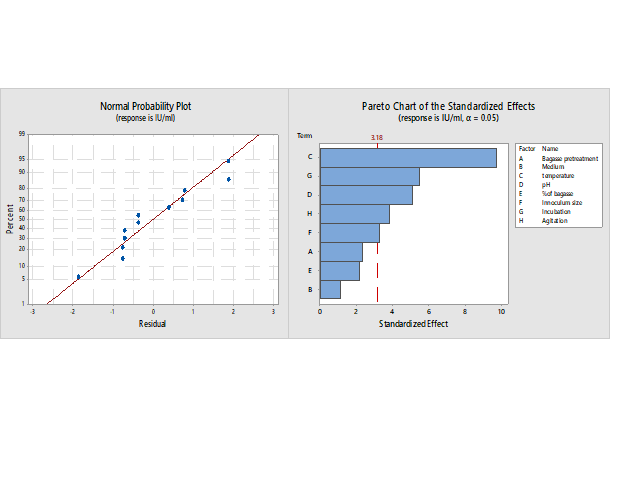


**Fig. S1** Pareto Chart of the standardized effects showing the significant factors


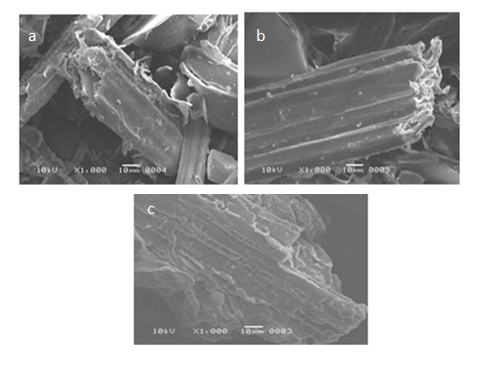


**Fig. S2** Scanning electron micrographs of various samples of SB (a) Untreated SB (b) NaOH+IL pretreated SB (c) Fermented SB with *Bacillus aestuarii* UE25
